# Developmental Differences in Gaze Behaviors and Performance During Basketball Free Throws in Youth Athletes

**DOI:** 10.3390/sports14030105

**Published:** 2026-03-06

**Authors:** Miaoyu Han, Carlos D. Gómez-Carmona, Daniele Conte, Jorge Lorenzo Calvo

**Affiliations:** 1 Facultad de Ciencias de la Actividad Física y del Deporte, Universidad Politécnica de Madrid, 28040 Madrid, Spain; jorge.lorenzo@upm.es; 2 Area de Educación Física y Deportiva, Departmento de Didáctica General y Didácticas Específicas, Facultad de Educación, Universidad de Alicante, 03690 San Vicente del Raspeig, Spain; 3 Grupo de Investigación Entrenamiento, Actividad Física y Rendimiento Deportivo (ENFYRED), Universidad de Zaragoza, 22001 Huesca, Spain; 4 Grupo de Investigación en Optimización del Entrenamiento y Rendimiento Deportivo (GOERD), Universidad de Extremadura, 10005 Caceres, Spain; 5 Department of Movement Human and Health Sciences, University of Rome “Foro Italico”, 00135 Rome, Italy; daniele.conte@uniroma4.it; 6 Department of Coaching Science, Lithuanian Sports University, 44221 Kaunas, Lithuania

**Keywords:** basketball, shooting performance, age-related, QE duration, talent development

## Abstract

(1) Background: This study investigated developmental differences in gaze behaviors and performance during basketball free throws among youth athletes. (2) Methods: Forty-six male youth basketball players (U14, U16, U18) each performed 30 standardized free throws while wearing Tobii Pro Glasses 3 to record gaze data (Quiet Eye duration and Total Fixation duration). Shooting accuracy and cognitive workload (NASA-TLX) were also collected. One-way ANOVA, Pearson correlation analysis, and multiple linear regression analysis were conducted to examine age-related differences and the relationships between gaze behavior and performance. (3) Results: Shooting accuracy was moderately correlated with chronological age (r = 0.386, *p* = 0.010) and training experience (r = 0.367, *p* = 0.010), and total fixation duration was positively associated with training experience (r = 0.338, *p* = 0.025). Regression analyses showed that training experience predicted total fixation duration, and both age and experience predicted shooting accuracy when considered separately (*p* < 0.05), but neither predicted cognitive workload (*p* > 0.05). Age and training experience were positively associated with shot success. (4) Conclusions: In the youth free-throw task, Quiet Eye duration and total fixation duration were highly correlated but did not independently predict shooting success, while shooting performance was more strongly associated with age and training experience, and perceived cognitive workload remained stable across age groups.

## 1. Introduction

In youth basketball, free-throw performance is considered a marker of attentional control, emotional regulation, and psychological maturity. Intervention studies indicate that training concentration and self-regulation can effectively improve free-throw accuracy and attentional stability in young players [1]. Within talent identification and development frameworks, psychological and perceptual–cognitive skills have been identified as important contributors to performance consistency under pressure [2,3]. As a self-paced precision task, the free throw relies on visual guidance, attentional control, and coordinated motor execution and therefore provides an appropriate context for examining these developmental capacities [2,4].

Gaze behavior plays a key role in perception–action coupling during free-throw performance by enabling athletes to acquire task-relevant visual information and control aiming and shot execution [4,5]. In accuracy-based tasks, performance stability depends not only on the mechanical consistency of motor execution but also on the maturity of visual control and attentional focus mechanisms that regulate perceptual–motor coordination [1,6,7]. These mechanisms allow performers to efficiently allocate visual attention, inhibit irrelevant stimuli, and maintain goal-directed focus, which distinguishes skilled performers from less experienced athletes [8,9]. For example, elite basketball players typically fixate on the hoop for longer periods prior to shot execution, and both the duration and frequency of these fixations have been identified as important determinants of shooting accuracy [10]. From a developmental perspective, gaze stability and attentional control reflect age- and skill-related adaptations associated with expertise acquisition [2].

Accordingly, gaze behavior during basketball free throws can be operationalized using established measures of visual attention and visuo-motor control. The ‘Quiet Eye’ (QE) is defined as the final fixation on a target prior to movement initiation and is associated with attentional stability and visuomotor coupling [4,11]. A longer QE duration is typically associated with more efficient attentional control and superior performance [5,12]. For example, professional basketball players typically exhibit a longer QE duration when making a free throw, which is significantly longer than that of novices [12,13,14,15]. In addition to QE duration, Total Fixation Duration (TFD) is defined as the cumulative duration of fixations directed toward the target, consistent with commonly applied fixation-based metrics in sport performance research [2,16]. Previous studies have shown that expert performers often demonstrate longer fixation durations on critical task-relevant areas compared to less-skilled athletes [2,16,17]. While QE captures the final fixation prior to movement initiation, TFD reflects the overall distribution of gaze toward the target across the execution phase [8]. Consistent with this conceptual framework, a recent systematic review by Alemanno et al. [18] synthesized current eye-tracking research in basketball and confirmed that expert players consistently exhibit longer QE durations and more efficient gaze strategies compared to novices. However, the review also highlighted several methodological limitations, particularly the limited ecological validity of many experimental paradigms and the scarcity of developmental comparisons [18].

The development of visual–motor coordination and executive functions during adolescence supports the maturation of attentional regulation and motor planning [6,19]. As athletes gain experience, they improve in filtering irrelevant stimuli, sustaining goal-directed focus, and efficiently integrating visual and motor information [20]. Nelson et al. [21] pointed out that during adolescence, executive control plays a central role in regulating motor behavior, health, and task performance. Around age 12, youth basketball players begin using adult-sized equipment and face adult-level visuomotor demands [22]. This developmental stage provides a unique context to study how perceptual–motor skills adapt to increasing task complexity. Examining gaze measures such as QE and TFD across adolescence can reveal how attentional control and motor planning co-develop [9,15,23].

Although numerous studies have examined gaze behaviors in adult or elite basketball players [24,25], systematic developmental comparisons remain limited [5]. In addition, much of the existing research has been conducted under controlled or semi-dynamic laboratory conditions, potentially restricting ecological validity and limiting insight into gaze allocation toward task-relevant targets in real performance contexts [9,10,12]. In parallel with theoretical developments in perceptual–cognitive research, recent methodological advances have integrated Artificial Intelligence (AI) and computer vision techniques with mobile eye-tracking systems to enable contextual gaze classification in ecologically valid basketball settings [26]. Such approaches allow dynamic target detection and real-time analysis of gaze behavior during live games, thereby enhancing ecological validity. However, controlled task paradigms remain essential for isolating fundamental developmental mechanisms. In self-paced free-throw tasks, the rim serves as the primary task-relevant visual target, providing a controlled context in which age-related differences in gaze allocation can be examined under standardized conditions. Around age 12, youth players transition to adult-sized equipment and regulation rim heights, creating a unique developmental window to examine how gaze allocation toward the rim adapts to increased task demands during adolescence [22,27,28]. By integrating both QE duration and TFD, the present study moves beyond simple performance to examine how fixation duration toward the rim and attentional stability vary across developmental stages as potential indicators of emerging expertise [27,28].

Therefore, this study aims to address this gap by examining age-related differences in gaze behavior, shooting accuracy, and cognitive workload across U14, U16, and U18 basketball players. We hypothesized that older youth basketball players (U18) would demonstrate significantly longer QE duration and TFD compared to younger age groups, and that age would positively correlate with gaze metrics and SA while negatively correlating with cognitive workload, potentially reflecting more efficient attentional allocation and lower perceived cognitive workload.

## 2. Materials and Methods

### 2.1. Participants

Forty-six male youth basketball players participated voluntarily in this study. An a priori power analysis was conducted using G*Power 3.1 for a one-way ANOVA with fixed effects (three groups). The analysis was based on an effect size of f = 0.40, an alpha level of 0.05, and a desired statistical power of 0.80. With the achieved sample size, the estimated statistical power was approximately 0.70 to detect large effects [29]. Although the achieved power was slightly below the conventional 0.80 threshold, it was considered acceptable for detecting large effects in exploratory developmental research, and effect sizes were therefore interpreted alongside *p*-values. Participants were recruited from Madrid Basketball Federation to ensure representation in different developmental levels and training systems. Among all participants, 44 were right-handed and 2 were left-handed shooters. All players had normal or corrected-to-normal vision and no history of neurological, visual, or musculoskeletal disorders. Due to injury and poor tracking quality (<20%), two participants were excluded from the final dataset. As a result, a complete statistical analysis was performed on 44 participants.

In Spanish youth basketball, age categories are organized in two-year competitive cycles according to the official regulations of the Spanish Basketball Federation (FEB), which define eligibility based on year of birth [30]. Data were collected at two time points: June 2025 (2024–2025 season; *n* = 28) and November 2025 (2025–2026 season; *n* = 16). Athletes assessed in November were newly recruited and had not participated in the June testing. Each participant contributed a single observation; therefore, the study employed a cross-sectional design. Both testing sessions were conducted during regular training periods and not immediately after competition. The final sample comprised: (1) Under 14 (U14) group (*n* = 14, age = 12.94 ± 0.43 years, range: 12.06–13.63 years, training experience = 5.50 ± 1.56 years); (2) Under 16 (U16) group (*n* = 15, age = 14.91 ± 0.43 years, range: 14.37–15.82 years, training experience = 7.20 ± 1.14 years); and (3) Under 18 (U18) group (*n* = 15, age = 16.70 ± 0.40 years, range: 16.16–17.77 years, training experience = 9.40 ± 1.30 years).

The study protocol was reviewed and approved by the Ethics Committee of the Faculty of Physical Activity and Sport Sciences (INEF), Universidad Politécnica de Madrid, and was conducted in accordance with the Declaration of Helsinki. Written informed consent was obtained from the parents or legal guardians of all participants prior to inclusion in the study, and verbal assent was obtained from the minor athletes.

### 2.2. Equipment

#### 2.2.1. Gaze Behaviors

Visual data were recorded using the Tobii Pro Glasses 3 (Tobii AB, Danderyd, Sweden), a wearable binocular eye-tracking system operating at 50 Hz. The device provides a spatial accuracy of approximately 0.6° and allows for natural head and body movements, making it suitable for dynamic sport tasks such as basketball free throws [31]. Previous research has shown that Tobii Pro Glasses 3 (Tobii AB, Danderyd, Sweden) exhibit high recording quality in mobile settings, with post-filtering data loss typically below 5% (for example, 0.12–3.07% across conditions) and precision and angular accuracy measures demonstrating consistent gaze tracking reproducibility [32,33].

#### 2.2.2. Cognitive Workload

Cognitive workload was assessed using the NASA Task Load Index (NASA-TLX) [34].

### 2.3. Experimental Procedure

**Figure 1:**
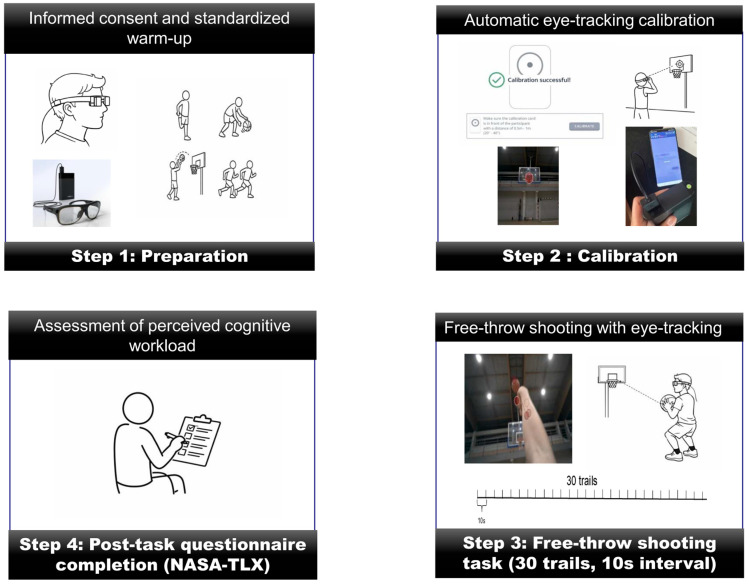
Overview of the experimental procedure.

All testing was conducted in an indoor basketball court meeting official FIBA standard, with a regulation rim height of 3.05 m and a free-throw line distance of 4.60 m. Participants used a standard size 7 basketball. Ambient lighting, background noise, and temperature were kept consistent across sessions. As shown in Figure 1, each participant’s testing session lasted approximately 30 min and included the following four stages:

1. Preparation: Participants completed a brief 5 min standardized basketball warm-up [35] consisting of light jogging, dynamic stretching and shooting from varied distances. They were then fitted with the Tobii Pro Glasses 3 eye-tracking system, which was connected to the recording unit.
2. Calibration: The Tobii Pro Glasses 3 system (Tobii AB, Danderyd, Sweden) was calibrated individually before each participant began the shooting task. Participants were fitted with the eye-tracking glasses and instructed to stand at the free-throw line while fixating steadily on the center of the basketball rim. Calibration was performed using the system’s built-in one-point automatic calibration procedure [31]. The participant-maintained fixation on the rim until the system emitted an auditory confirmation signal indicating successful calibration and stable gaze tracking. Calibration quality was visually verified on an OPPO smartphone connected to the Glasses 3 to ensure that the gaze cursor was accurately aligned with the rim location in the scene view. If noticeable spatial deviation or unstable tracking was observed, the calibration procedure was repeated. Each participant underwent calibration immediately prior to testing to minimize potential drift due to head movement or repositioning of the glasses.
3. Shooting: Each participant performed 30 consecutive free throws from the regulation free-throw line. A 10 s rest interval was allowed between throws to reduce fatigue and maintain concentration [4,24]. Gaze data were recorded continuously by the Tobii Pro Glasses 3 while shot outcome (hit/miss) was manually recorded by the researcher.
4. Questionnaire completion: Following completion of the shooting task, participants completed the NASA Task Load Index (NASA-TLX) questionnaire to assess perceived cognitive workload.

### 2.4. Dependent Variable Assessment

Gaze data were processed using Tobii Pro Lab software (version 25.19.1151, Tobii AB, Danderyd, Sweden), which enabled fixation detection and the computation of key visual metrics. Fixations were initially identified using the default I-VT (Velocity-Threshold Identification) algorithm implemented in Tobii Pro Lab (version 25.19.1151, Tobii AB, Danderyd, Sweden) (minimum duration = 100 ms; velocity threshold = 30°/s) [32,36]. No additional filtering or smoothing procedures were applied beyond the default Tobii Pro Lab settings. Binocular gaze data were used, and fixation classification was based on the averaged gaze position provided by the software. QE duration and TFD were manually annotated frame-by-frame within the timeline view to ensure precise temporal alignment with the rim area of interest (AOI) and movement events.

**Figure 2:**
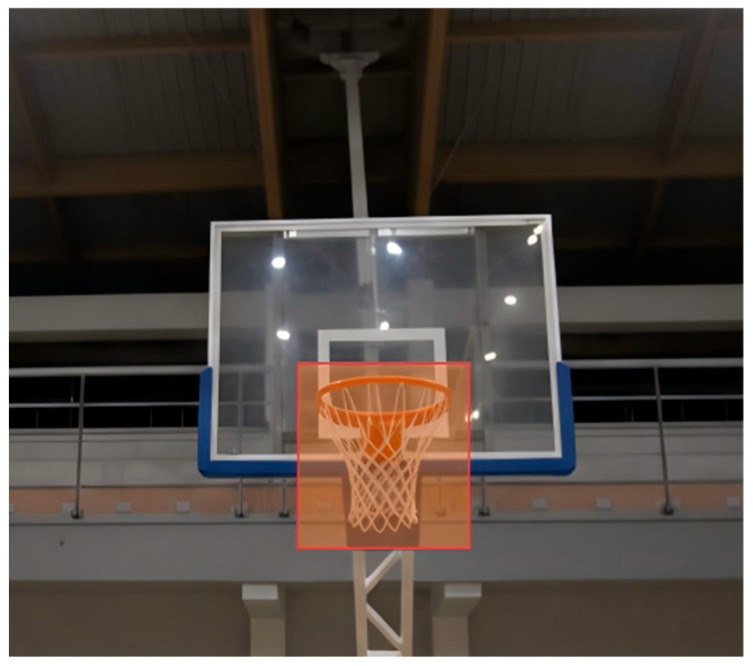
Area of interest (AOI) defined around the basketball rim.

The rim AOI was defined as a square dynamic AOI centered on the rim for each recording using Tobii Pro Lab’s frame-by-frame mapping function (Figure 2). The AOI boundaries were adjusted to account for minor head movements to ensure accurate fixation classification. The AOI radius was kept constant across participants to ensure comparability.

Movement initiation was identified through frame-by-frame inspection of the synchronized scene camera video within Tobii Pro Lab. The onset of the shooting movement was operationally defined as the first observable and continuous upward elevation of the shooting arm preceding ball release. This frame was cross-checked using the synchronized timeline view displaying gaze horizontal and vertical angles to ensure accurate temporal alignment between motor and visual events.

QE duration was defined as the final fixation or tracking gaze that is located on a specific object or location within 3° of visual angle for a minimum of 100 ms before the initiation of the final movement [4]. QE onset corresponded to the first frame of the final continuous fixation within the rim AOI before movement initiation, and QE offset was defined as the last consecutive frame before gaze exited the rim.

TFD was defined as the cumulative duration of all fixation segments directed toward the rim area of interest (AOI) during each shooting trial, consistent with fixation-based metrics commonly applied in sport eye-tracking research [2,16,24]. TFD accumulation began at the first fixation within the rim AOI and ended at the frame corresponding to ball release. Multiple fixation episodes occurring within the AOI were included. TFD values were calculated by summing fixation segments within the AOI. Only fixation samples classified within the predefined rim AOI were included in the calculation. Samples marked as invalid by the software (e.g., due to blink or temporary signal loss) were excluded, and no interpolation was applied.

Ball-release detection was determined through frame-by-frame inspection of the scene camera video within Tobii Pro Lab. The release frame was verified by synchronizing the scene camera view with gaze timeline data to ensure temporal alignment between motor execution and gaze behavior. The moment of ball release was operationally defined as the first frame in which the ball was no longer in contact with the shooting hand.

Gaze offset for QE duration was defined as the first frame in which fixation samples were located outside the rim AOI for more than two consecutive frames (≥40 ms at 50 Hz). Brief single-frame deviations were not considered offset. If tracking was lost due to blink or signal dropout, QE offset was defined as the last valid fixation frame within the AOI prior to data loss. TFD did not rely on a single continuous fixation segment but represented the total time gaze was directed toward the rim prior to ball release. Brief interruptions (e.g., blinks or temporary signal loss) were excluded from calculations, and no interpolation was applied.

Invalid gaze samples caused by blinks or tracking loss were automatically flagged by Tobii Pro Lab and manually verified. Trials containing more than 20% invalid gaze samples were excluded from analysis. On average, 27.4 ± 1.9 valid trials per participant were retained for analysis, and gaze metrics were averaged across valid trials to obtain a single representative value per participant.

Missing data rate was calculated at the participant level as the proportion of invalid gaze samples relative to the total number of recorded samples. Specifically, missing rate (%) was computed as (Invalid samples/Total samples) × 100. The overall mean missing data rate across participants was 4.8 ± 1.2%. A one-way ANOVA revealed no significant differences in missing data proportions between age groups (*p* > 0.05), indicating that data loss was not systematically associated with developmental level.

Each free-throw attempt was coded as a binary outcome (hit = 1, miss = 0). For participant-level analyses, shooting accuracy was calculated as the percentage of successful shots across valid trials. Finally, overall cognitive workload scores were calculated using the Raw NASA-TLX approach, defined as the mean of the six subscales (mental demand, physical demand, temporal demand, performance, effort, and frustration), each rated from 0 to 100.

### 2.5. Statistical Analysis

All statistical analyses were conducted with IBM SPSS Statistics 29.0.2.0 (IBM Corp., Armonk, NY, USA). The normality of data distribution was assessed using the Shapiro–Wilk test. To account for potential seasonal effects, testing month (June vs. November) was examined as an additional factor in preliminary analyses. No significant main or interaction effects of testing month were observed for any dependent variable (*p* > 0.05); therefore, data were pooled for subsequent analyses.

Differences between age groups (U14, U16, U18) in QE duration, TFD, SA, and NASA-TLX workload scores were analyzed using one-way ANOVA, with Tukey’s HSD post hoc tests applied where appropriate. A post hoc power analysis indicated acceptable power for detecting medium effects. Effect sizes for ANOVA were reported as partial eta squared (η^2^). According to Cohen [37], η^2^ values below 0.06 indicate a small effect, values between 0.06 and 0.14 indicate a medium effect, and values greater than 0.14 indicate a large effect. Training experience differed significantly across age groups (F_(2, 41)_ = 30.997, *p* < 0.001), necessitating covariate adjustment. ANCOVA was performed with training years as a covariate to examine whether age-group effects remained significant after controlling for practice exposure. Statistical significance was set at *p* < 0.05.

To address the developmental continuity of athletes, chronological age was also examined as a continuous predictor. Bivariate relationships between age, experience, gaze measures, and performance were assessed using Pearson correlation coefficients (r). To reduce the risk of inflated Type I error due to multiple correlation analyses, the Benjamini–Hochberg false discovery rate (FDR) correction (α = 0.05) was applied to correlation results.

Prior to regression modeling, multicollinearity was rigorously assessed; a correlation threshold of r > 0.80 was considered indicative of problematic redundancy, in which case predictors were entered into separate models to preserve coefficient stability. Additionally, formal diagnostics were conducted where Variance inflation factors (VIF) > 5 and Tolerance values < 0.20 served as exclusion criteria. Predictors exceeding these thresholds were modeled independently to ensure the statistical integrity of the results.

A two-stage regression approach was employed to evaluate predictive power. First, simple linear regression models estimated the independent contributions of age and training experience. Second, multiple linear regression models were implemented where age and experience were entered simultaneously to examine their unique variance while controlling for inter-correlation. Finally, to account for the repeated-trial structure (30 trials per participant), a Generalized Linear Mixed Model (GLMM) was implemented at the trial level. Each attempt (hit = 1, miss = 0) was modeled using a binomial distribution with a logit link function. Participant No. was entered as a random intercept, while gaze behaviors, age, and experience were fixed effects. For all regression and GLMM models, unstandardized coefficients (B), standard errors (SE), and 95% confidence intervals (CI) were reported to indicate the direction and precision of the estimates. In the GLMM, Odds Ratios (OR) were further calculated to interpret the likelihood of shooting success relative to gaze metrics.

## 3. Results

### 3.1. Age-Group Differences in Gaze Behaviors and Performance

Table 1 presents age-group differences in gaze behavior, shooting accuracy, and cognitive workload. Results indicated that no significant main effect of age group on QE duration, F_(2, 41)_ = 1.847, *p* = 0.170, η^2^ = 0.083, or on TFD, F_(2, 41)_ = 1.915, *p* = 0.160, η^2^ = 0.085. Although the U18 group exhibited numerically higher mean QE duration and TFD values compared with the U14 and U16 groups, these differences were not statistically significant. The corresponding effect sizes were in the medium range.

SA increased across age groups, although the overall group effect did not reach statistical significance F_(2, 41)_ = 2.620, *p* = 0.085, η^2^ = 0.113, reflecting a medium effect size.

Cognitive workload (NASA-TLX) scores showed a slight decrease with increasing age-group, but no significant group differences were observed, F_(2, 40)_ = 0.940, *p* = 0.399, η^2^ = 0.044, indicating a small effect size.

After controlling for training experience, the main effect of age-group remained non-significant for all outcome variables: QE duration, F_(2, 40)_ = 1.892, *p* = 0.164, partial η^2^ = 0.086; TFD, F_(2, 40)_ = 1.946, *p* = 0.156, partial η^2^ = 0.089; SA, F_(2, 40)_ = 0.379, *p* = 0.687, partial η^2^ = 0.019; and cognitive workload, F_(2, 40)_ = 0.271, *p* = 0.764, partial η^2^ = 0.013. Effect sizes were in the medium range for QE duration and TFD and small for SA and cognitive workload.

### 3.2. Relationships Between Gaze Behaviors and Performance

Table 2 showed the Pearson correlations between age, training experience, gaze variables, SA, and cognitive workload. A very strong positive correlation was observed between QE duration and TFD (*r* = 0.938, *p* < 0.001). Age was strongly correlated with training experience (*r* = 0.820, *p* < 0.001).

SA showed moderate positive correlations with both age (*r* = 0.386, *p* = 0.010) and training experience (*r* = 0.367, *p* = 0.010). TFD showed positive correlations with training experience (*r* = 0.338, *p* = 0.025). QE duration was moderately correlated with training experience (r = 0.292), although this association did not reach statistical significance (*p* = 0.054).

In contrast, QE duration was not significantly associated with age (*r* = 0.089, *p* = 0.564) or SA (*r* = 0.142, *p* = 0.357), and TFD was not significantly correlated with SA (*r* = 0.044, *p* = 0.775), while no significant correlations were observed between cognitive workload (NASA-TLX) and age, training experience, gaze behavior, or SA (all *p* > 0.05).

### 3.3. Predictive Power of Gaze Behavior Metrics on Performance

Multicollinearity diagnostics revealed substantial redundancy between QE and TFD, with VIF values of 8.414 and 8.852 and Tolerance values of 0.119 and 0.113, respectively. These findings align with their high Pearson correlation (*r* = 0.938, *p* < 0.001). Consequently, QE and TFD were analyzed in independent regression models to preserve coefficient stability.

In contrast, age and training experience demonstrated acceptable multicollinearity levels, with VIF values (3.365 and 3.762, respectively) and Tolerance values (0.297 and 0.266, respectively) both within the conservative threshold of 5. These findings indicate that while age and experience are strongly related (r = 0.820, *p* < 0.001), their shared variance does not violate the assumptions of the mixed-effects models, allowing for their simultaneous inclusion to independently control for biological maturation and specialized training duration.

#### 3.3.1. Age and Training Experience Models

Table 3 showed linear regression models predicting gaze behavior, SA, and cognitive workload from age and training experience. Age was not significantly associated with QE duration or TFD (*p* > 0.05). Training experience was significantly associated with TFD (*p* = 0.025), whereas its association with QE duration did not reach statistical significance (*p* = 0.054).

Both age (*p* = 0.010) and training experience (*p* = 0.014) showed significant positive association with SA. No significant associations were observed between age or training experience and cognitive workload (*p* > 0.05).

Table 4 showed chronological age and training experience as simultaneous predictors. Training experience remained a significant predictor of QE duration (β = 0.667, *p* = 0.011), whereas age was not significant (*p* = 0.076). For TFD, both age (β = −0.499, *p* = 0.048) and training experience (β = 0.747, *p* = 0.004) were significant predictors.

For SA, although the overall model was significant (*p* = 0.030), neither chronological age nor training experience independently predicted performance when entered simultaneously (*p* > 0.05). The model predicting cognitive workload was not significant (*p* = 0.476).

#### 3.3.2. Trial-Level Predictive Models

Results across all models consistently demonstrated that developmental factors (age and experience), demonstrated stronger statistical associations of trial-level success.

For QE duration, the overall effects were significant for both the training experience model (F_(2, 1298)_ = 3.343, *p* = 0.036) and the chronological age model (F_(2, 1298)_ = 3.490, *p* = 0.031). Training experience significantly predicted a higher likelihood of success (B = 0.160, SE = 0.062, *p* = 0.010, OR = 1.174, 95% CI [1.038, 1.326]), indicating that each additional year of experience increased the odds of a successful shot by 17.4%. Similarly, chronological age was a robust positive predictor (B = 0.211, SE = 0.080, *p* = 0.009, OR = 1.235, 95% CI [1.055, 1.445]), corresponding to a 23.5% increase in the odds of success per year. Notably, QE duration did not significantly predict outcomes in either model (*p* > 0.05).

Consistent findings emerged from the models involving TFD The overall model effects remained significant when paired with age (F_(2, 1297)_ = 3.32, *p* = 0.037), or experience (F_(1, 1297)_ = 3.13, *p* = 0.044). Both age (F_(1, 1297)_ = 6.62, *p* = 0.010, OR = 1.235) and experience (F_(1, 1297)_ = 6.25, *p* = 0.013, OR = 1.163) remained significant positive predictors, while TFD showed no independent association with shot success (*p* > 0.05).

When chronological age and training experience were entered simultaneously as fixed effects, neither predictor retained statistical significance (*p* > 0.05)

## 4. Discussion

This study investigated age-related differences in gaze behavior, shooting accuracy, and cognitive workload across U14, U16, and U18 basketball players. The results showed no significant age-group differences in QE duration, TFD, SA, or perceived cognitive workload, and these findings remained unchanged after controlling for training experience. SA was moderately correlated with both age and training experience, whereas TFD was positively associated with training experience but not with SA. Cognitive workload was not related to age, experience, gaze measures, or performance. These findings suggest that perceived workload remained relatively stable across age groups within the context of a self-paced free-throw task. Regression analyses indicated that training experience significantly predicted TFD, and both age and training experience predicted shooting accuracy when examined separately. However, neither variable independently predicted SA when entered simultaneously. In the multiple regression models, training experience was significantly associated with TFD, whereas chronological age was not.

### 4.1. Age-Related Differences

Contrary to our hypothesis, age-group classification did not significantly differentiate gaze behavior or free-throw performance. Although effect sizes for QE duration and total fixation duration were moderate, the differences were not statistically significant and remained non-significant after controlling for training experience [37].

Previous research has reported longer QE durations in experts compared with novices [4,17], whereas developmental comparisons within youth samples are less consistent. The strong correlation between chronological age and training experience indicates that these variables are closely related, which complicates the interpretation of age as an independent explanatory factor [23,38,39].

Although adolescence is generally characterized by ongoing development in cognitive control and working memory [40], the present cross-sectional design does not permit inference regarding individual developmental trajectories [3,7,40]. From an expertise development perspective, accumulated training experience has been associated with increased stability in well-practiced motor tasks [41]. In the present study, no statistically significant differences were observed in NASA-TLX scores across age groups, nor were workload scores significantly associated with age, training experience, gaze metrics, or shooting accuracy, indicating relative stability in perceived effort within this task context [34]. Basketball free throws represent uncontested and self-paced motor actions, which typically impose lower external attentional demands than dynamic game situations involving defensive pressure and temporal constraints [42]. Tasks characterized by high predictability and temporal self-regulation typically elicit stable subjective workload ratings [8,22,42]. Accordingly, the absence of workload differences may reflect the low cognitive complexity of the experimental task [24,34].

### 4.2. Gaze–Performance Relationship

A strong positive correlation was observed between QE duration and total fixation duration, suggesting substantial construct overlap between the two measures. This extremely high correlation suggests substantial construct overlap between these metrics. Specifically, QE duration was defined as the final fixation prior to movement initiation directed toward the rim [4,27], whereas TFD represented the cumulative fixation time within the rim AOI before ball release [43,44]. Previous research has suggested that sustained visual coupling during both preparation and execution phases may contribute to successful motor performance in aiming tasks [8,12,42]. In basketball contexts, stable visual alignment with the target has been associated with improved shooting consistency under certain conditions [24,45].

Chronological age was not significantly associated with QE duration or TFD, indicating that age alone accounted for minimal variance in visual control measures within this sample. Rather than reflecting a linear developmental improvement, this finding suggests that age-related differences in visuomotor control may be subtle and potentially mediated by other factors such as task experience or perceptual–cognitive efficiency. Older athletes may appear more capable of filtering irrelevant stimuli and maintaining goal-directed visual focus [46,47]. However, given the limited strength of the observed association, such age-related trends may be only partially related to the maturation of the fronto-parietal attention network and the gradual automatization of attentional control [17,48]. From a performance perspective, such improvements in sustained visual control are particularly relevant in basketball free throws, as free throws are uncontested, self-paced scoring opportunities that frequently occur during decisive phases of competition [1,2]. In addition, research has highlighted that stable visual attention and efficient visuomotor coupling are key components distinguishing higher-level performers and are increasingly recognized as markers of long-term athletic potential rather than isolated performance outcomes [2,23].

In contrast, QE duration did not correlate significantly with shooting accuracy, indicating that fixation duration alone was insufficient to explain shooting performance variance in this youth sample [9,15,17]. This finding differs from expert–novice comparisons reporting longer QE durations in higher-performing athletes [4,17], suggesting that gaze–performance relationships may be less pronounced within relatively homogeneous youth samples [9]. In youth athletes, longer fixation durations may reflect compensatory control strategies rather than efficient visuomotor integration [9,15]. Moreover, the relationship between QE duration and shooting accuracy may be moderated by contextual factors such as competitive pressure, physical fatigue, or task constraints, all of which have been shown to influence visuomotor control and attentional regulation during basketball shooting [15,24]. In real-game situations, free throws often occur under elevated pressure or accumulated physical load, conditions under which youth athletes may not yet be able to consistently translate stable gaze behavior into successful motor execution.

### 4.3. Predictive Value of Gaze Behavior and Performance

Regression analyses revealed different patterns at the participant and trial levels, clarifying the relative contributions of age, training experience, and gaze behavior to shooting outcome.

At the participant level, training experience was significantly associated with TFD, whereas chronological age was not significantly related to QE duration or TFD. Expertise research has consistently emphasized the importance of accumulated practice in shaping performance-related behaviors [49], and sport-specific perceptual–cognitive research similarly suggests that training history may account for variability in task-relevant visual strategies [2,3]. Accordingly, the present findings indicate that sustained TFD was statistically associated with practice exposure rather than chronological age alone. The cross-sectional design does not allow conclusions regarding directionality between age and training experience. Rather, the results indicate that within this sample, training experience accounted for more variance in TFD than age when examined independently.

With respect to SA, both age and training experience were significant predictors when examined separately in linear regression models. However, when entered simultaneously, neither predictor retained statistical significance. This attenuation is consistent with the strong shared variance between age and training experience, suggesting that their individual associations with performance were not statistically independent. From a regression modeling perspective, highly correlated predictors can substantially reduce each other’s unique explanatory contribution due to multicollinearity, even when each demonstrates significant effects in isolation [37]. In youth sport contexts, chronological age and accumulated training exposure are typically related, as older athletes often have more years of structured practice. As athletes mature, increases in age are typically accompanied by greater cumulative practice exposure, structured coaching, and competitive experience [38]. Expertise research further emphasizes that deliberate practice, rather than age alone, underlies skill acquisition and performance refinement [3,49]. Accordingly, gaze duration may facilitate motor preparation but does not independently determine performance outcomes in low-pressure, self-paced youth contexts.

This study found neither QE duration nor TFD significantly predicted SA at the participant level. This finding contrasts with research demonstrating longer QE durations in higher-skilled performers [17,27]. However, many prior investigations have compared clearly differentiated skill groups (e.g., experts vs. novices) or examined performance under elevated task demands [2,11,15]. Within the present relatively homogeneous youth cohort performing a low-complexity, self-paced task, fixation duration alone may not sufficiently capture the multidimensional determinants of successful performance. Within a relatively homogeneous youth sample, variability in performance may reflect multiple interacting factors beyond fixation duration alone.

Cognitive workload was not significantly predicted by age or training experience in either single- or multi-predictor models. This statistical stability suggests that perceived effort remained relatively constant across individuals within the present task context. Previous sport psychology research has indicated that subjective workload ratings may show limited variability in predictable, self-paced motor tasks. In the absence of external time pressure or defensive constraints, individual differences in demographic or experiential variables may exert minimal influence on perceived cognitive demand.

Trial-level generalized linear mixed models further supported this pattern. Chronological age and training experience significantly predicted the probability of shot success when modeled separately, whereas QE duration and TFD did not independently predict trial-level outcomes. This consistency across analytical levels reinforces the conclusion that demographic and experiential variables demonstrated stronger associations with performance than gaze duration metrics within this task context. However, when age and experience were entered simultaneously as fixed effects, neither retained statistical significance, again reflecting their substantial shared variance.

Under such stable and predictable task constraints, performance variability may be more strongly associated with accumulated experiential factors than with moment-to-moment gaze duration variability [50,51]. In the present study, the task (uncontested free throw) and environment were highly stable and predictable. Under such constrained conditions, individual experiential factors may exert greater influence than moment-to-moment visual engagement variability. Accordingly, gaze duration may play a facilitative role in motor preparation but may not independently determine performance outcomes in low-pressure, self-paced youth contexts.

Overall, these findings indicate that in adolescent athletes performing a controlled free-throw task, accumulated training exposure and chronological age are associated with performance, whereas QE duration and TFD do not independently predict success. These findings contribute to ongoing discussions regarding the boundary conditions of QE duration effects [52,53].

### 4.4. Practical Implications

The present findings provide preliminary insights into the relative associations between age, training experience, gaze behavior, and shooting performance in a controlled free-throw context.

Training experience demonstrated stronger statistical associations with sustained TFD and shooting success than gaze duration metrics alone within a controlled free-throw context. Accordingly, practical emphasis may be placed on structured and accumulated task-specific practice exposure, which showed consistent associations with both visual control behavior and performance outcomes.

Given that neither QE duration nor TFD independently predicted shooting accuracy at the participant or trial level, fixation duration should not be interpreted as a standalone performance determinant in similar youth free-throw settings. Instead, gaze behavior should be considered one component alongside technical execution, motor consistency, and accumulated training exposure.

Furthermore, perceived cognitive workload did not significantly differ across age groups and was not predicted by age, training experience, or gaze measures. This relative stability suggests that in predictable and uncontested task conditions, subjective effort may remain consistent across adolescent athletes.

### 4.5. Limitations and Future Research Directions

This study has several limitations. First, the sample size was relatively modest and included only male athletes, which may limit the generalizability of the findings.

Second, the experimental protocol involved a controlled block of 30 uncontested free throws performed under standardized conditions. The low-complexity and predictable nature of this task may partly explain the absence of significant gaze–performance associations. Free throws are performed from a young age in basketball. Although it is an individual, closed-skill task, the competitive context in which it is performed is complex and multifactorial. Moreover, stress-regulation components should not be limited to U18 training, as gaze behavior and cognitive-load regulation are still developing across earlier age groups. A practical approach is to simulate game-like physiological fatigue by administering free throws after short sprints/running bouts or dribbling drills.

Although this design ensured methodological consistency and reduced contextual variability, it does not fully reflect the dynamic, competitive, and psychologically demanding nature of real-game situations. The absence of defensive pressure, time constraints, score-related stress, and environmental distractions may limit the ecological and external validity of the findings. Therefore, caution is warranted when generalizing these results to in-game performance contexts.

Third, the cross-sectional design prevents causal inference and does not allow determination of whether the observed associations between age, training experience, and performance reflect developmental processes or cohort effects. In addition, the study did not include anthropometric measures (e.g., height, arm span, body mass) or indicators of biological maturation (e.g., peak height velocity). Given the substantial inter-individual variability in biological maturation during adolescence, the absence of these measures may have contributed to unexplained variability in gaze and performance outcomes [41,54]. Future studies may incorporate objective maturation indicators to clarify the potential influence of biological maturation on gaze behavior and performance associations.

Future studies should adopt longitudinal designs to track intra-individual changes across adolescence and apply multimodal measures—combining eye tracking with EEG, pupillometry, or heart-rate variability—to obtain complementary physiological and cognitive indicators. In addition, expanding the sample to include female athletes and testing interventions in ecologically valid contexts (e.g., during competitive games) would improve both the robustness and applicability of the findings. Furthermore, advances in mobile eye-tracking and computer vision technologies may facilitate more ecologically valid assessments of gaze behavior in dynamic sport environments.

## 5. Conclusions

This study examined age-related associations in gaze behavior, shooting accuracy, and perceived cognitive workload during basketball free throws among adolescent athletes. No statistically significant age-group differences were observed in QE duration, TFD, shooting accuracy, or perceived cognitive workload.

At the participant level, chronological age and training experience were positively associated with shooting accuracy in single-predictor models; however, these effects did not remain statistically independent when entered simultaneously. Training experience showed a statistically significant association with TFD in single-predictor models. Neither QE duration nor TFD independently predicted shooting success at the participant or trial level.

The strong correlation between QE duration and TFD indicates substantial construct overlap between these measures within the present task context. Additionally, perceived cognitive workload remained statistically stable across age groups and was not predicted by age, training experience, or gaze behavior.

In general, these findings suggest that within a controlled and self-paced youth free-throw task, accumulated training exposure and chronological age demonstrated stronger statistical associations with performance outcomes than fixation duration alone. Future work should test these relationships under more ecologically valid constraints (e.g., fatigue/pressure) and may benefit from AI-supported eye-tracking pipelines to improve contextual interpretation of gaze behavior.

## Figures and Tables

**Table 1:** Descriptive statistics (Mean ± SD) of gaze behavior, SA, and cognitive workload by age group.

Variable	U14 (*n* = 14)	U16 (*n* = 15)	U18 (*n* = 15)
QE duration (ms)	359.97± 198.60	284.76 ± 195.66	478.33 ± 386.34
TFD (ms)	704.06 ± 315.61	560.25 ± 235.64	852.08 ± 583.34
SA (%)	48.57 ± 23.60	60.22 ± 18.66	64.22 ± 14.00
NASA-TLX (scores)	47.79 ± 12.89	46.98 ± 8.98	42.86 ± 9.31

QE duration = Quiet Eye duration, TFD = Total fixation duration, SA = shooting accuracy, NASA-TLX = NASA Task Load Index (cognitive workload), U14 = Under 14 years old, U16 = Under 16 years old, U18 = Under 18 years old.

**Table 2:** Pearson correlations between Age, Experience, QE, TFD, SA and Cognitive Workload.

Variable	Age (Years)	Experience (Years)	QE (ms)	TFD (ms)	SA (%)
Experience (years)	0.820 *p* < 0.001	--	--	--	--
QE (ms)	0.089 *p* = 0.564	0.292 *p* = 0.054	--	--	--
TFD (ms)	0.113 *p* = 0.465	0.338 *p* = 0.025	0.938 *p* < 0.001	--	--
SA (%)	0.386 *p* = 0.010	0.367 *p* = 0.010	0.142 *p* = 0.357	0.044 *p* = 0.775	--
NASA-TLX	−0.116 *p* = 0.455	−0.180 *p* = 0.242	−0.091 *p* = 0.556	−0.145 *p* = 0.347	−0.022 *p* = 0.886

Experience = Training experience, QE = Quiet-eye duration, TFD = Total fixation duration, SA= Shooting accuracy, NASA-TLX = NASA Task Load Index (cognitive workload), *p* < 0.05 considered significant. “--” indicates self-correlations and duplicated values. All significant results remained significant after Benjamini–Hochberg FDR correction.

**Table 3:** Linear regression models predicting gaze behavior, SA, and cognitive workload from age and training experience.

Dependent Variable	Predictor	β	SE	t	*p*	Adjusted R^2^
QE duration	Age	0.089	27.181	0.582	0.564	−0.016
	Experience	0.292	20.089	1.978	0.054	0.063
TFD	Age	0.113	27.181	0.738	0.465	−0.011
	Experience	0.338	29.225	2.324	0.025	0.093
SA	Age	0.386	1.762	2.715	0.010	0.129
	Experience	0.367	1.368	2.554	0.014	0.114
Cognitive Workload (NASA-TLX)	Age	−1.116	1.005	−0.754	0.455	−0.01
	Experience	−0.18	0.766	−1.186	0.242	0.009

QE duration = Quiet Eye duration, TFD = Total fixation duration, SA = shooting accuracy, Cognitive workload (NASA-TLX) = NASA Task Load Index (scores), *p* < 0.05 considered significant. β represents standardized regression coefficients; SE refers to unstandardized standard errors. All significant results remained significant after Benjamini–Hochberg FDR correction.

**Table 4:** Multiple regression models including chronological age and training experience as simultaneous predictors.

Dependent Variable	Predictor	β	SE	t	*p*
QE duration	Age	−0.458	44.389	−1.823	0.076
	Experience	0.667	34.165	2.658	0.011
Model fit	Adj. R^2^ = 0.113, *p* = 0.033				
TFD	Age	−0.499	63.969	−2.041	0.048
	Experience	0.747	49.232	3.053	0.004
Model fit	Adj. R^2^ = 0.156, *p* = 0.012				
SA	Age	0.262	3.101	1.046	0.302
	Experience	0.152	2.387	0.606	0.328
Model fit	Adj. R^2^ = 0.116, *p* = 0.030				
Cognitive Workload (NASA-TLX)	Age	0.09	1.758	0.365	0.717
	Experience	−0.26	1.353	−0.971	0.337
Model fit	Adj. R^2^ = −0.011, *p* = 0.476				

QE duration = Quiet Eye duration, TFD = Total fixation duration, SA = shooting accuracy, Cognitive workload (NASA-TLX) = NASA Task Load Index (scores), *p* < 0.05 considered significant. β represents standardized regression coefficients; SE refers to unstandardized standard errors. All significant results remained significant after Benjamini–Hochberg FDR correction.

## Data Availability

Data are available from the corresponding author upon request.

## Abbreviations

The following abbreviations are used in this manuscript:

QE	Quiet Eye
TFD	Total Fixation duration
SA	Shooting Accuracy
NASA-TLX	NASA Task Load Index (cognitive workload)
AI	Artificial Intelligence
U14	Under 14 years old
U16	Under 16 years old
U18	Under 18 years old
VIF	Variance inflation factors
GLMM	Generalized linear mixed model
FDR	Benjamini–Hochberg false discovery rate
